# CD73 is associated with glycolysis related metabolic programs and CD8^+^ T cell suppression in pancreatic ductal adenocarcinoma

**DOI:** 10.3389/fimmu.2026.1873813

**Published:** 2026-07-27

**Authors:** Eriko Katsuta, Aarohan Mukherjee Burma, Eito Nakagawa, Ken Oh, Tao Dai, Subhamoy Dasgupta, Kazuaki Takabe, Daisuke Ban

**Affiliations:** 1 Department of Hepatobiliary and Pancreatic Surgery, Graduate School of Medical and Dental Sciences, Institute of Science Tokyo, Tokyo, Japan; 2 Department of Cell Stress Biology, Roswell Park Comprehensive Cancer Center, Buffalo, NY, United States; 3 Department of Surgical Oncology, Roswell Park Comprehensive Cancer Center, Buffalo, NY, United States; 4 Department of Surgery, University at Buffalo Jacobs School of Medicine and Biomedical Sciences, The State University of New York, Buffalo, NY, United States

**Keywords:** CD73, glycolysis, hypoxia, immune suppression, pancreatic ductal adenocarcinoma

## Abstract

CD73 is an enzyme that generates extracellular adenosine and has been implicated in tumor-associated immune suppression, but its biological and clinical significance in pancreatic ductal adenocarcinoma (PDAC) remains incompletely understood. We investigated the prognostic relevance and biological functions of CD73 in PDAC using transcriptomic analyses, *in vitro* functional assays, and *in vivo* mouse models. Transcriptomic analyses consistently showed that CD73 expression was correlated with hypoxia, glycolysis related, and cell-cycle programs. In agreement with these findings, hypoxic exposure induced CD73 mRNA expression in a subset of PDAC cell lines, and CD73 knockdown modestly affected glycolysis related extracellular acidification (ECAR) in a cell line dependent manner, and modulation of CD73 expression altered PDAC cell growth. CD73 expression was also associated with reduced intratumoral CD8^+^ T cell infiltration and cytolytic activity in human PDAC cohorts. *In vivo*, CD73 overexpression accelerated cancer progression and shortened survival in immunocompetent mice, whereas this effect was abrogated in immunodeficient NSG mice. Flow cytometric analysis further demonstrated reduced intratumoral CD8^+^ T cell infiltration in CD73 overexpressing tumors. Clinically, high CD73 expression was consistently associated with worse survival in multiple PDAC cohorts. Collectively, these findings suggest that CD73 is associated with multiple features of PDAC progression, including hypoxia-related metabolic programs, tumor growth, and suppression of anti-tumor immunity. CD73 may therefore represent a biologically relevant biomarker and a potential therapeutic target in PDAC.

## 1 Introduction

Pancreatic ductal adenocarcinoma (PDAC) remains one of the most aggressive cancers with a poor prognosis. Although systemic therapy has advanced in recent years, the prognosis remains poor, with a 5-year survival rate of approximately 13% (1, 2). Therefore, identifying biologically relevant and therapeutically actionable molecular pathways is crucial in PDAC.

CD73, also known as ecto-5′-nucleotidase, catalyzes the conversion of extracellular adenosine monophosphate (AMP) to adenosine, in concert with CD39, which hydrolyzes adenosine triphosphate (ATP) and adenosine diphosphate (ADP) to AMP (3–5). Recent studies and reviews have highlighted the CD39/CD73 purinergic signaling axis as a central mechanism of immune evasion in cancer, particularly through adenosine-mediated suppression of effector lymphocytes and remodeling of myeloid and stromal compartments (6). In PDAC, this pathway is particularly relevant because the tumor microenvironment is characterized by hypoxia, dense desmoplasia, myeloid-rich inflammation, and profound resistance to immunotherapy (7). Adenosine signal is activated mainly through A2A and A2B receptors (8, 9). A2A receptor signaling is a dominant suppressor of anti-tumor lymphocyte function and inhibits CD8^+^ T cell and natural killer (NK) cell cytotoxicity largely through cAMP/PKA-mediated suppression of TCR-driven effector programs. In parallel, A2B receptor signaling, which becomes particularly relevant under high-adenosine conditions, promotes myeloid-dominant immunosuppression by enhancing suppressive antigen-presenting cell and myeloid cell programs. Together, these pathways shift the tumor microenvironment toward impaired effector immunity and enhanced immune tolerance (8, 10–12). In PDAC, major components of the adenosine pathway include CD39 and CD73, which generate extracellular adenosine, together with downstream signaling through A2A and A2B receptors. Previous reports indicate that CD39 is enriched mainly in tumor-infiltrating myeloid cells (13), whereas CD73 is expressed by both cancer cells and infiltrating immune cells (13, 14), particularly myeloid populations. In addition, A2A receptor expression has been reported predominantly in tumor-infiltrating myeloid populations in PDAC models (14), whereas A2B receptor expression has been described across multiple immune and stromal compartments in the PDAC tumor microenvironment (15). In addition, CD73 is reported to be overexpressed in multiple types of cancer (16–19). High expression of CD73 is associated with cancer invasiveness and metastasis in leukemia and prostate cancer (20, 21), and shows worse survival in colorectal cancer (22), gastric cancer (23) and gallbladder cancer (24). On the other hand, there are conflicting reports that high expression of CD73 is associated with either better or worse prognosis in some types of cancer, including breast cancer (25, 26) and ovarian cancer (27, 28). Previously, we have discovered that CD73 was upregulated in pancreatic neuroendocrine tumor initiating cells and related to their invasiveness (29).

In PDAC, the tumor microenvironment is profoundly hypoxic, desmoplastic, and immunosuppressive (30). These features suggest that pathways involved in immune evasion and tumor adaptation to a hostile microenvironment may play important roles in disease progression. Recently, cancer cell CD73 and adenosine metabolism was shown to contribute to immunosuppression in PDAC in a tumor-autonomous and autocrine manner (31). Building on this evidence, we aimed to determine whether CD73 is also associated with hypoxia-related metabolic programs, cancer cell growth, and CD8^+^ T cell suppression. This study therefore extends the current understanding of CD73 in PDAC by integrating transcriptomic analyses, *in vitro* functional assays, *in vivo* mouse models, and clinical survival analyses to evaluate the metabolic, immunologic, and prognostic relevance of CD73.

## 2 Materials and methods

### 2.1 Cell culture and CD73 modulation

Human normal pancreatic epithelial cell line, HPDE, and human pancreatic cell lines, AsPC-1, BxPC-3, DAN-G, KLM-1, MIAPaCa-2, PANC-1, and PSN-1 were obtained from ATCC (Manassas, VA, USA), murine pancreatic cancer cell line, Panc02 was obtained from NCI DCTD Tumor Repository. HPDE, MIAPaCa-2, DAN-G, PANC-1, PSN-1, Panc02 were cultured in Dulbecco’s Modified Eagle Medium (DMEM) (Sigma-Aldrich, St. Louis, MO, USA) with 10% fetal bovine serum (FBS) (SRN, Pessin, Germany) and 1% Pen/Strep (Thermo Fisher, Waltham, MA, USA), AsPC-1, BxPC-3, KLM-1 were cultured in RPMI (Sigma-Aldrich) with 10% FBS and 1% Pen/Strep. All cell cultures were routinely tested to rule out mycoplasma infection using MycoStrip (InvivoGen, San Diego, CA, USA). All cell lines were cultivated in a humidified incubator at 37 °C in 5% CO_2_. For hypoxia experiments, cells were exposed to hypoxic conditions (1% O_2_, 5% CO_2_) in a chamber for 24 h using a BioSpherix Xvivo System. The oxygen concentration inside the chamber was continuously monitored by the system and confirmed on the digital display during hypoxic exposure. Successful activation of the hypoxic response was further assessed by western blot of Hypoxia-inducible factor-1α (HIF-1α) and quantitative PCR for canonical HIF-1α-responsive genes, including *GLUT1* and *PDK1*. Panc02 cells were transfected with *luc2* plasmid (Promega; pGL4.20 [*luc2*/Puro] Vector) using Lipofectamine LTX with PLUS reagent (Invitrogen, Thermo Fisher) according to the manufacturer’s instructions and selected with 2 µg/mL puromycin for at least 2 weeks (Gibco, Thermo Fisher) to establish stable cells. For overexpression experiments, Panc02 cells were transfected with an empty vector or a mouse CD73 expression plasmid (Origene; empty vector PS100001, mCD73 MR227439) using Lipofectamine LTX with PLUS reagent (Invitrogen, Thermo Fisher) according to the manufacturer’s instructions and selected with 500 µg/mL G418 for at least 2 weeks (Gibco, Thermo Fisher) to establish stable overexpression cells. For knockdown experiments, AsPC-1 and PANC-1 cells were transduced with shNT (non-targeting) or shCD73 (Clone ID: pGIPZ V2LHS 152370) and selected with 2 µg/mL puromycin for at least 2 weeks (Gibco, Thermo Fisher) to establish stable knockdown cells. Cell proliferation was assessed using a Cell Counting Kit-8 (CCK-8; Dojindo, Kumamoto, Japan). Cells were seeded into 96-well plates at 1,000 cells/well (n=6) and cultured for 4 consecutive days. CCK-8 reagent was added to each well and incubated for 3 h at 37 °C, after which absorbance was measured at 450 nm wavelength to quantify viable cells.

### 2.2 RNA extraction and quantitative real-time PCR

Total RNA was extracted using RNeasy Mini Kit (Qiagen, Venlo, Netherlands) from cells cultured under normoxic or hypoxic conditions and reverse-transcribed into cDNA using High-Capacity cDNA Reverse Transcription Kit (Applied Biosystems, Foster City, CA, USA). Quantitative real-time PCR (qPCR) was carried out using a real-time PCR system with gene-specific primers for *CD73*, *GLUT1*, and *PDK1*. Primer sequences were listed in **Supplementary Table 1**. Gene expression levels were normalized to *ACTB*, as commonly used in pancreatic cancer qPCR studies (32, 33), and relative mRNA expression was calculated using the comparative ^ΔΔ^Cq method. Results are presented as relative expression levels compared with normoxic controls.

### 2.3 Seahorse extracellular flux assay

Extracellular acidification rate (ECAR) was measured using a Seahorse XFe96 Analyzer (Agilent, Santa Clara, California). Cells were seeded in Seahorse XF96 cell culture microplates (Agilent) at 4 × 10^4^ cells/well. On the day of the assay, growth medium was replaced with pre-warmed Seahorse XF assay medium (pH 7.4 at 37 °C) supplemented with 2 mM L-glutamine without glucose or pyruvate, and cells were incubated at 37 °C in a non-CO_2_ incubator for 45–60 min prior to measurement. The ECAR glycolysis stress test was performed with sequential injections of glucose (final 10 mM), oligomycin (final 1 μM), and 2-deoxyglucose (2-DG; final 50 mM). Each measurement cycle consisted of 3 min mixing and 3 min measurement. ECAR values are presented as mpH/min/1000 cells, normalized to cell number as shown in the figure.

### 2.4 Western blotting

Cells were lysed with RIPA buffer (Invitrogen, Thermo Fisher), and lysates were separated by electrophoresis and transferred onto a nitrocellulose membrane. Membranes were blocked with 5% milk for 1 h at room temperature and then incubated with primary antibody (CD73; 1:1,000, from Cell Signaling Technology (Danvers, MA), HIF-1α; 1:1000, from Cell Signaling Technology, Actin; 1:40,000 from Millipore Sigma (Burlington, MA)) at 4 °C overnight. Blots were developed with HRP-labelled secondary antibodies, followed by Clarity Western ECL detection system (BioRad, Hercules, CA). Chemiluminescence signals were acquired using a ChemiDoc MP imager (BioRad).

### 2.5 Animal care and use

The animal study protocol was approved by Roswell Park Comprehensive Cancer Center. A PDAC peritoneal carcinomatosis model was conducted using Panc02-*luc2* cells. NSG and C57BL/6 (male, 6–8 weeks-old) mice were purchased in-house from Roswell Park Comprehensive Cancer Center. 1×10^6^ Panc02-*luc2* cells, of either stable empty vector (CTRL) or CD73 (CD73-OE) transfected, in 1 mL of PBS were inoculated intraperitoneally into the mice. For survival experiments, tumor burden was quantified by IVIS bioluminescence imaging regularly, and an animal was euthanized once an animal reached humane endpoint. At the humane endpoint, mice were euthanized by CO_2_ inhalation using a gradual-fill method, with a CO_2_ flow rate corresponding to 30–70% of the chamber volume per minute, in accordance with the approved institutional animal care protocol. Another set of syngeneic experiments was conducted for immune analysis. 15 days after tumor inoculation, the tumors were harvested and processed for immune cell analysis.

### 2.6 Immune cell quantifying

Harvested tumors were dissociated into a single cell suspension using a Tumor Dissociation Kit (Miltenyi Biotec, Bergisch Gladbach, Germany) and the gentleMACS dissociator (Miltenyi Biotec) as per manufacturer instructions. Tumors were then incubated in a Hybaid incubator (Phoenix Equipment) for 1 h. The resulting mixture was strained through 100-μm SureStrain cell strainers (MTC Bio). The single cell suspensions were treated by Fc block and then stained with antibodies and measured by LSRFortessa (BD). Antibodies are as follows: CD3-APC (Biolegend, San Diego, CA, USA), CD4-PE/Cy7 (Biolegend), CD8a-FITC (Biolegend), CD11b-BV510 (Biolegend), Gr-1-PE (Biolegend). CD4^+^ T cells were defined as CD3^+^ and CD4^+^, CD8^+^ T cells were defined as CD3^+^ and CD8^+^. CD11b^+^Gr-1^+^ myeloid population was defined as CD3^-^, CD11b^+^ and Gr-1^+^ cells.

### 2.7 T cell proliferation assay

CD8^+^ T cells were collected from C57BL/6 mouse spleen. Briefly, harvested spleen was smashed and strained through SureStrain cell strainers, red blood cells (RBC) was lysed with ACK lysing buffer (Gibco, Thermo Fisher), and CD8^+^ T cells were isolated using Mojosort magnet (Biolegend) as per manufacturer’s instruction. Isolated CD8^+^ T cells were stained with CFSE (Invitrogen, Thermo Fisher) and resuspend into TIL media (AIM-V (Thermo Fisher) 45 mL, FBS 5 mL, 2-mercaptoethanol (Millipore-Sigma) 0.175 μL) with IL-2 (R&D). 5×10^4^ of either Panc02 control cells or CD73 overexpression cells were seeded into 24-well plate the day before T cell adding. 5×10^5^ of CD8^+^ T cells were added into each well and CD3CD28 dynabeads (Gibco, Thermo Fisher) were added to stimulate T cells as per manufacturer’s protocol. After 3 days incubation, beads were removed by placing on the magnet and T cells were stained with CD8-APC (Biolegend) antibody and live-dead aqua (ThermoFisher), then fixed with 4% paraformaldehyde (PFA). CFSE were analyzed by FACSCalibur (BD, New Jersey, USA).

### 2.8 Data acquisition and pre-processing

The gene expression level quantification data (mRNA expression z-score from RNA sequencing) for The Cancer Genome Atlas (TCGA) pancreatic adenocarcinoma (PAAD) cohort were downloaded through cBioPortal (34) and used as previously described (35, 36). The TCGA PAAD cohort included 179 patients, of whom OS and DFS data were available for 177 and 138 patients, respectively. As a validation cohort, we obtained paired gene expression and survival profiles of 288 and 215 PDAC samples from E-MTAB-6134 and ICGC-CA, respectively. Based on the previous study (37), patients were classified into CD73-high and CD73-low groups using the upper tertile as the cutoff. The same cutoff strategy was applied across all cohorts to avoid dataset-specific optimization and improve reproducibility.

### 2.9 GSEA of TCGA cohort

To identify biological pathways associated with CD73 expression, gene set enrichment analysis (GSEA) was performed using a correlation-based approach. Briefly, normalized gene expression data were used to calculate the correlation between CD73 expression and the expression of all other genes across samples. Spearman’s rank correlation coefficient was used as the ranking metric. Genes were then ordered from the most positively correlated to the most negatively correlated genes according to the correlation coefficient, generating a preranked gene list. GSEA was subsequently performed using the GSEAPreranked module in GSEA software (Broad Institute).

### 2.10 Statistical analysis

Survival analyses were conducted using Kaplan-Meier curves with log-rank test. Continuous variables between two groups were compared using Student’s t-test or the Wilcoxon rank-sum test, as appropriate. Immune cell composition in the TCGA cohort was estimated using the CIBERSORT algorithm (38) applied to bulk RNA-seq data. Relative fractions of 22 immune cell populations were compared between CD73-high and CD73-low tumors using the Wilcoxon rank-sum test. The CD8 score was defined as the mean expression of CD8A and CD8B (39), the cytolytic activity (CYT) score was calculated as the geometric mean of GZMA and PRF1 expression (40), the a T-cell exhaustion (EXH) score was defined as the mean expression of established T-cell exhaustion markers, including PDCD1, HAVCR2, LAG3, TIGIT, and TOX (41). Differences between CD73-high and CD73-low tumors were compared using the Wilcoxon rank-sum test. For exploratory analyses involving multiple immune-related signatures or cell populations, P values were interpreted cautiously because these analyses were hypothesis-generating. When applicable, multiple testing was addressed using the Benjamini–Hochberg false discovery rate method. In all analyses, a two-sided p<0.05 was considered as statistically significant. All statistical analyses were performed using GraphPad Prism (GraphPad, San Diego, CA, USA) and R software (http://www.r-project.org/) together with Bioconductor (http://bioconductor.org/).

## 3 Results

### 3.1 CD73 is highly expressed in pancreatic ductal adenocarcinoma

To characterize the landscape of CD73 expression across cancer types, we analyzed pan-cancer transcriptomic profiles from the TCGA database, which revealed high *CD73* expression in PDAC compared with many other tumor types (**Supplementary Figure 1A**). *CD73* mRNA expression in PDAC ranked among the higher tiers of tumor types analyzed, highlighting its potential biological relevance in this highly aggressive malignancy. Next, we investigated CD73 protein level of normal pancreatic epithelium and several pancreatic cancer cell lines. CD73 was not expressed in HPDE that was derived from normal pancreatic epithelium, whereas CD73 was expressed in all other examined PDAC cells with variable expression levels (**Supplementary Figure 1B**).

### 3.2 CD73 transcription is enhanced by hypoxia in PDAC

**Figure 1:**
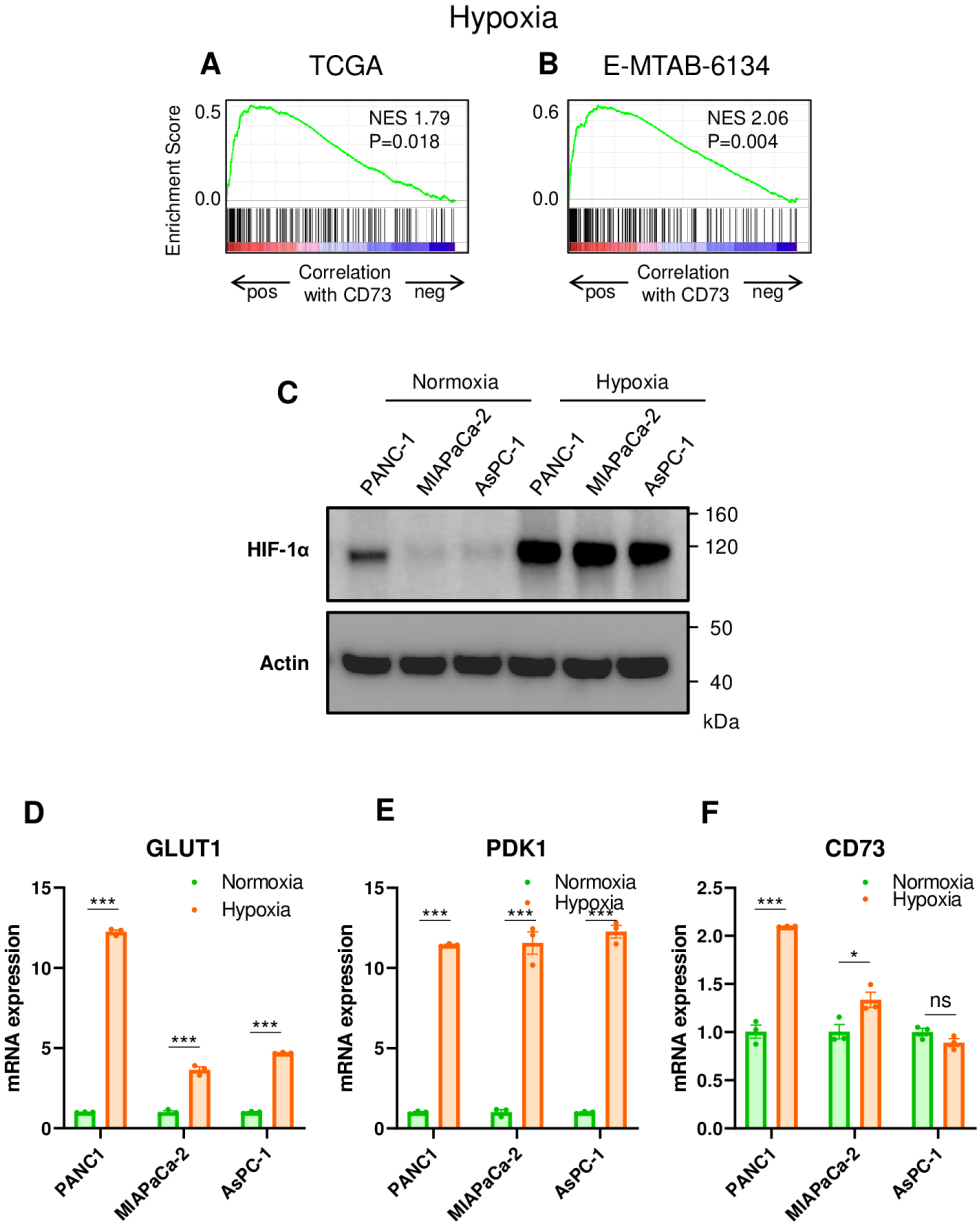
CD73 expression is associated with hypoxia related signature in pancreatic ductal adenocarcinoma (PDAC). Gene set enrichment analysis (GSEA) of hypoxia correlation with CD73 in **(A)** TCGA and **(B)** E-MTAB-6134 cohorts. PDAC cell lines, PANC-1, MIAPaCa-2 and AsPC-1 were cultured in normoxia (21% O_2_) or hypoxia (1% O_2_). **(C)** Immunoblotting shows the levels of HIF-1α in normoxic and hypoxic conditions. mRNA expression of **(D)** GLUT1, **(E)** PDK1, and **(F)** CD73 under hypoxia or normoxia (n=3). ***P<0.001, **P<0.01, *P<0.05.

Previous studies have shown that Hypoxia-inducible factor-1α (HIF-1α) directly binds to the *CD73* promoter and transcriptionally upregulates *CD73* expression in normal epithelial cells and gastric cancer cells (4, 42). To determine whether this regulatory axis is also present in PDAC, we first assessed the association between hypoxia-related gene signatures and *CD73* expression across two independent PDAC cohorts. Gene set enrichment analysis (GSEA) revealed a significant positive correlation between *CD73* and hypoxia signatures in the TCGA and E-MTAB-6134 cohorts (**Figures 1A, B**). In order to determine whether hypoxia causes *CD73* transcription in PDAC cells, we cultured PDAC cells under hypoxic conditions. To verify activation of the hypoxia pathway, we first confirmed HIF-1α protein stabilization under hypoxic conditions by western blot (**Figure 1C**). Consistent with this finding, we then measured the mRNA levels of established HIF-1α target genes, *GLUT1* and *PDK1* (43, 44), where both were robustly upregulated under hypoxia (**Figures 1D, E**). We then confirmed hypoxic condition increased *CD73* mRNA expression in two out of the three tested PDAC cell lines (**Figure 1F**), supporting that CD73 is inducible under hypoxic stress and is likely regulated by HIF-1α in PDAC.

### 3.3 CD73 is associated with glycolytic metabolic programs in PDAC

**Figure 2:**
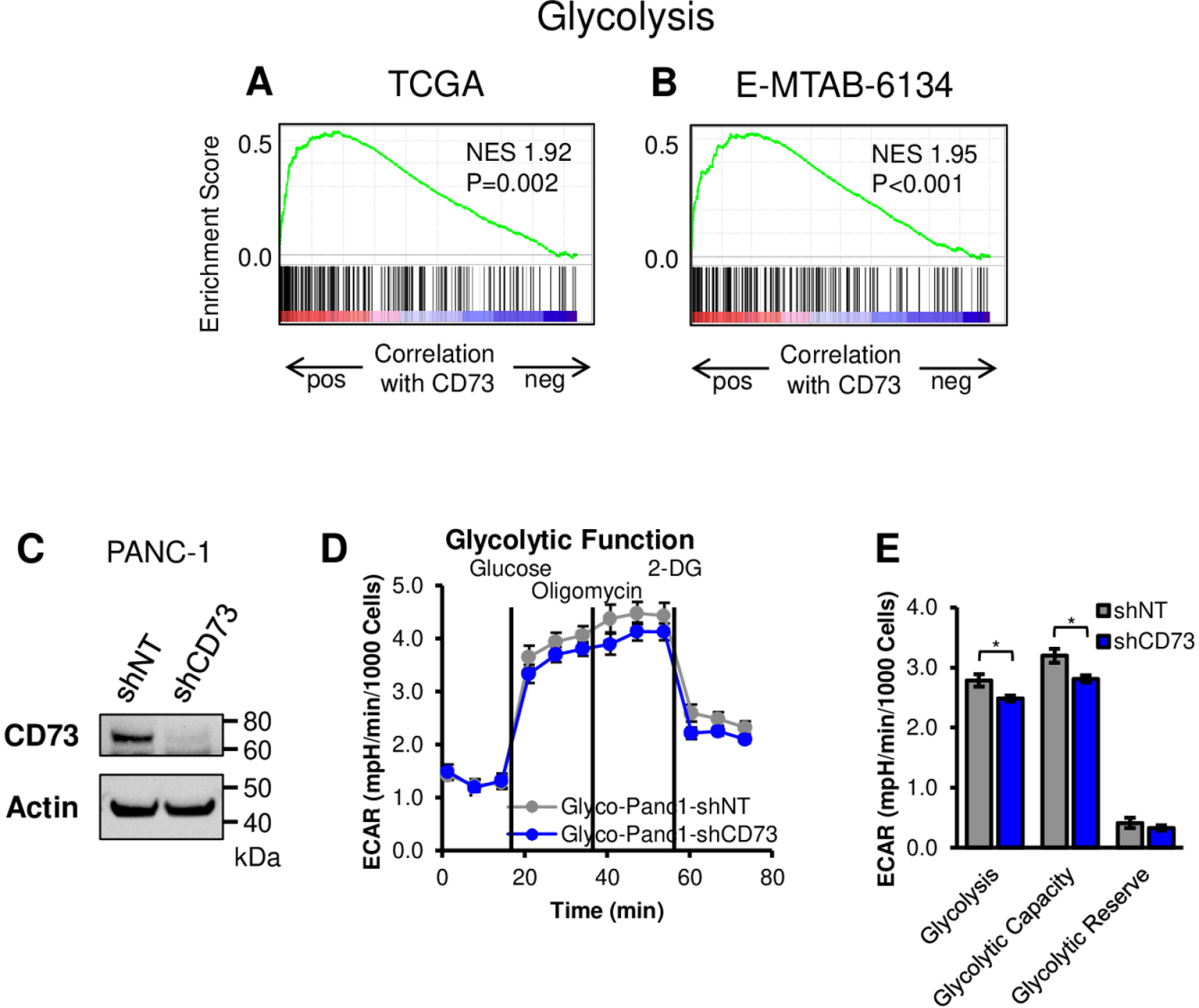
CD73 is associated with glycolysis-related metabolic activity in pancreatic ductal adenocarcinoma (PDAC). GSEA of glycolysis correlation with CD73 in **(A)** TCGA and **(B)** E-MTAB-6134. **(C)** Establishment of CD73 knockdown PANC-1 cell. **(D, E)** Glycolysis function change in CD73 knockdown PANC-1 cells by Seahorse assay (n=6). ***P<0.001, **P<0.01, *P<0.05.

Given that CD73 is transcriptionally enhanced under hypoxic conditions and hypoxia is a well-established driver of metabolic reprogramming (42), we next examined whether CD73 is associated with glycolysis related metabolic programs in PDAC. GSEA across two independent cohorts consistently demonstrated a strong positive correlation between *CD73* expression and glycolysis-related genes (**Figures 2A, B**). In order to functionally assess whether CD73 contributes to glycolysis related metabolic activity, we established a CD73 stable knockdown PANC-1 cells (**Figure 2C**). CD73 knockdown in PANC-1 cells modestly reduced ECAR following glucose and oligomycin treatment by Seahorse extracellular flux analysis (**Figures 2D, E**). However, a similar effect was not clearly observed in AsPC-1 cells (**Supplementary Figure 2**), indicating that the metabolic impact of CD73 may be cell line dependent. Taken together, these findings suggest that CD73 is associated with glycolysis related metabolic programs in PDAC and may contribute to glycolysis-related metabolic regulation in context dependent manner.

### 3.4 CD73-driven glycolytic reprogramming enhances cell-cycle progression in PDAC

**Figure 3:**
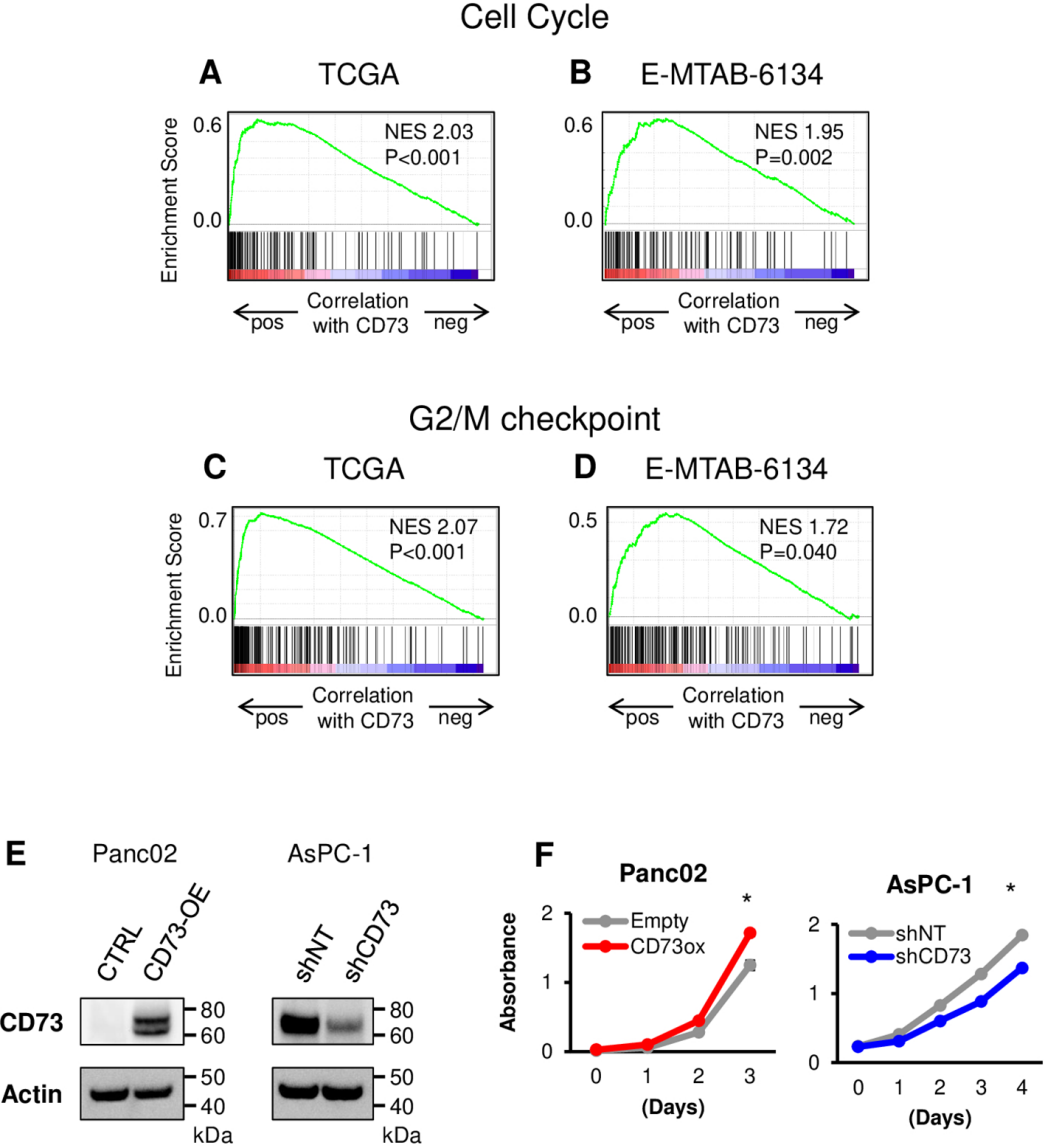
CD73 is associated with cell cycle related programs and altered pancreatic ductal adenocarcinoma (PDAC) cell growth. GSEA of cell cycle in **(A)** TCGA and **(B)** E-MTAB-6134. GSEA of G2/M checkpoint in **(C)** TCGA and **(D)** E-MTAB-6134. **(E)** Establishment of stable CD73 overexpression Panc02 and CD73 knockdown AsPC-1. **(F)** Cell proliferation assay of CD73 overexpressed Panc02 and CD73 knockdown AsPC-1 (n=6). ***P<0.001, **P<0.01, *P<0.05.

Because glycolysis is tightly linked to cell cycle progression by supplying biosynthetic substrates and regulatory metabolites (45, 46), we next investigated whether CD73-driven glycolytic activation translates into increased proliferative capacity in PDAC cells. GSEA demonstrated that *CD73* expression was consistently associated with enriched cell-cycle signatures in the TCGA and E-MTAB-6134 datasets (**Figures 3A, B**). Moreover, gene sets related to the G2/M checkpoint were also correlated with *CD73* expression (**Figures 3C, D**), supporting an association between CD73 expression and a transcriptional signature consistent with heightened cell cycle activity. In addition, pathways related to nucleotide excision repair and p53 signaling were also correlated to CD73 expression in both cohorts (**Supplementary Figure 3**), further suggesting that CD73 expression is linked to broader programs involved in cell cycle regulation and cellular stress responses. To determine whether these transcriptomic features translate into altered cancer cell growth, we established CD73-overexpressing PDAC cells using CD73 low expressing Panc02 cells, as well as CD73-knockdown cells using AsPC-1 cells and confirmed efficient modulation of CD73 expression by western blotting (**Figure 3E**). CD73 overexpression significantly increased the proliferation of Panc02 cells, whereas CD73 knockdown reduced the proliferation of AsPC-1 cells (**Figure 3F**). Taken together, these findings support the notion that CD73 contributes to PDAC progression by promoting glycolysis-associated proliferative programs and cancer cell growth.

### 3.5 High CD73 expression PDAC is associated with reduced CD8^+^ T-cell infiltration in human PDAC

**Figure 4:**
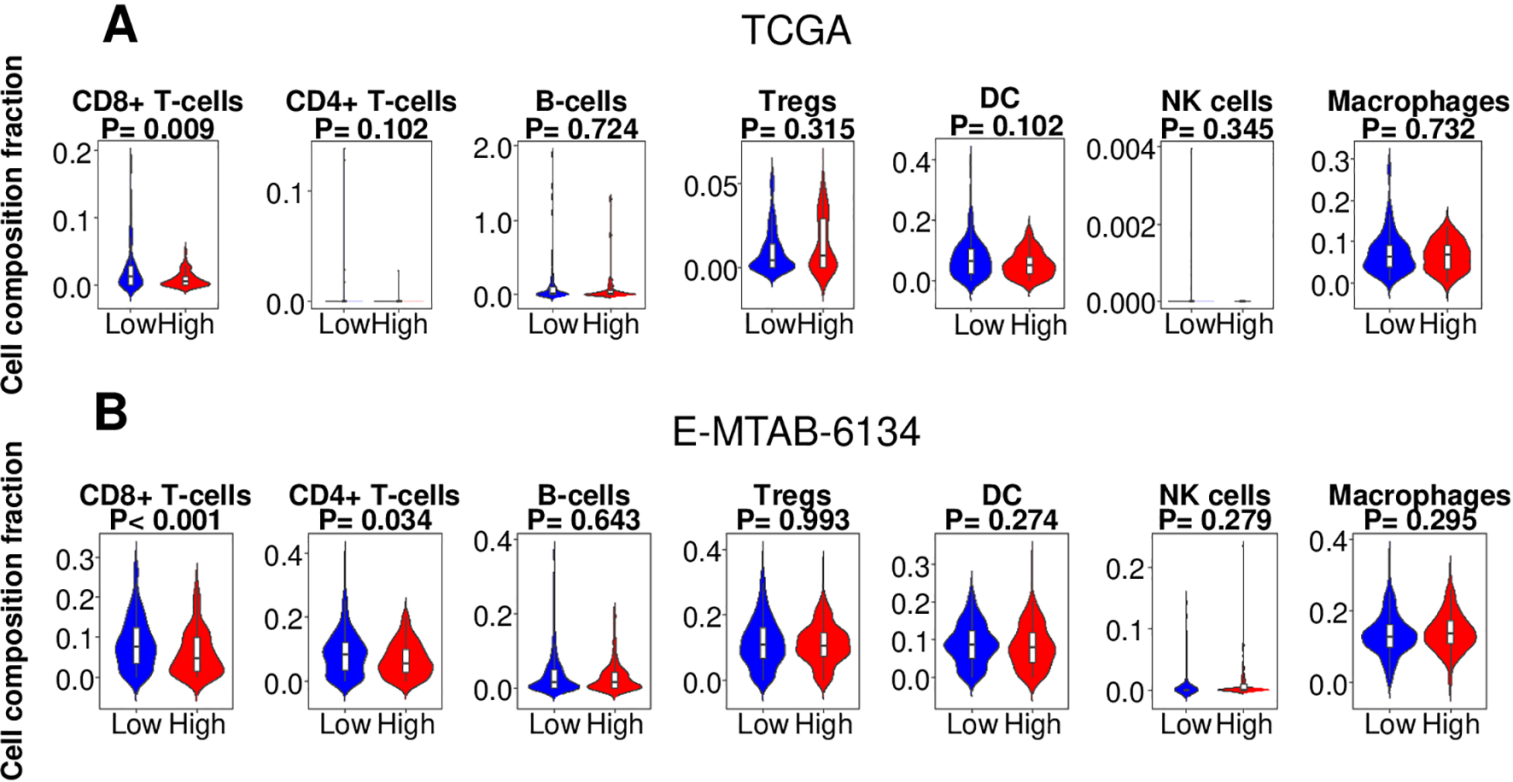
CD73 high expressing tumor showed significantly less CD8^+^ T cell infiltration in pancreatic ductal adenocarcinoma (PDAC) human cohort. The association of CD73 with immune cells in PDAC. Cell composition fraction estimated by Cibersort algorithm in **(A)** TCGA and **(B)** E-MTAB-6134.

Since CD73 is a key ectonucleotidase that converts AMP to adenosine (3, 4), and adenosine is known to suppress anti-tumor immunity by impairing effector T cell function and promoting an immunosuppressive tumor microenvironment (8, 47), we next examined immune cell infiltration by *CD73* expression in human PDAC samples. In the TCGA dataset, *CD73*-high tumor displayed a significantly lower fraction of infiltrating CD8^+^ T cells compared with *CD73*-low tumor (**Figure 4A**). A similar reduction in CD8^+^ T-cell abundance in *CD73*-high tumor was observed in the E-MTAB-6134 cohort (**Figure 4B**), supporting the association across datasets. Interestingly, CD73-high tumors exhibited significantly lower CD8 T cell and cytolytic activity scores, whereas exhaustion-related signatures were not significantly different between the groups (**Supplementary Figure 4**). Because these scores were derived from bulk RNA-seq data, exhaustion-related gene expression is influenced by the overall abundance of infiltrating T cells. Therefore, the absence of an increased exhaustion score does not exclude the possibility that residual T cells in CD73-high tumors may have an exhausted phenotype. Rather, these findings suggest that the predominant immune feature of CD73-high PDAC is reduced CD8^+^ cytotoxic T cell infiltration and cytolytic activity, rather than a clearly exhaustion-dominant transcriptomic pattern. On the other hand, other reported immune cell subsets influenced by extracellular adenosine, including CD4^+^ T cells, B cells, dendritic cells, NK cells, and macrophages, did not consistently differ between *CD73*-high and *CD73*-low tumors. Collectively, these findings suggest that elevated CD73 expression in PDAC is associated with a more immunosuppressed tumor microenvironment, characterized by reduced CD8^+^ T cell infiltration.

### 3.6 CD73 enhances tumor growth *in vivo* with suppressed CD8^+^ T cells

**Figure 5:**
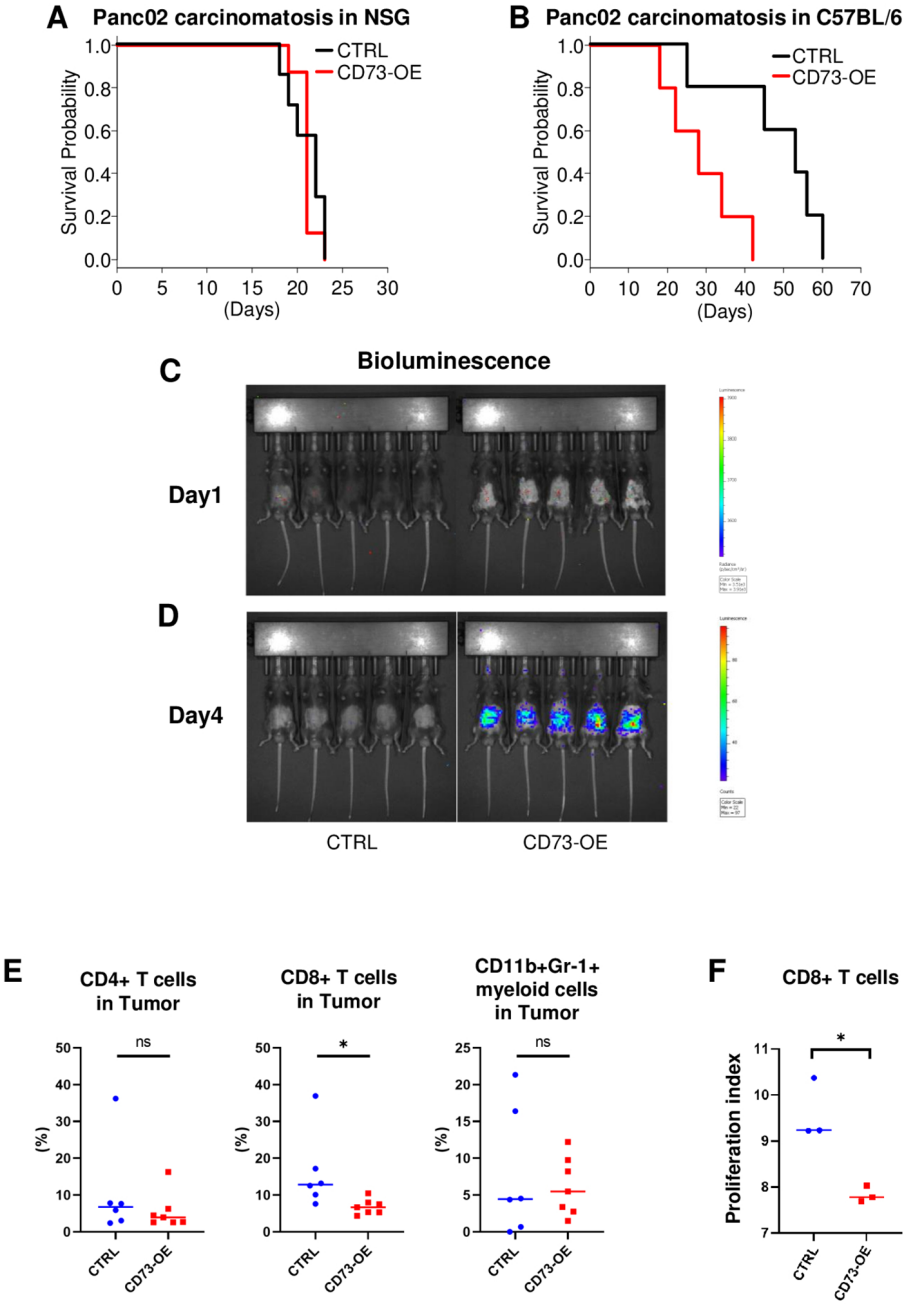
CD73 promotes cancer growth in syngeneic, but not in immune deficient mice carcinomatosis model of pancreatic ductal adenocarcinoma (PDAC). **(A)** Survival curve of CD73 overexpressed Panc02 carcinomatosis in immune deficient NSG mice (n=7, each). **(B)** Survival curve of CD73 overexpressed Panc02 carcinomatosis in syngeneic C57BL/6 model. n=5, each). Bioluminescence of CD73 overexpressed Panc02 carcinomatosis model in syngeneic model on Day 1 **(C)** and on Day 4 **(D)**. **(E)** Infiltrating immune cells into the tumors in syngeneic model (CTRL; n=6, CD73-OE; n=7). **(F)** CD8^+^ T cell proliferation assay co-cultured with CD73-overexpressing Panc02 cells. ***P<0.001, **P<0.01, *P<0.05.

To determine whether CD73 induced immune suppressive environment promotes tumor progression *in vivo*, we used Panc02 carcinomatosis models. Intraabdominal injection of CD73 overexpressing cells into immune deficient NSG mice showed no survival difference compared to control cells (**Figure 5A**). In contrast, CD73 overexpressing carcinomatosis exhibited significantly shorter survival compared to control in immune competent C57BL/6 mice (**Figure 5B**). As expected, overall survival in NSG mice was shorter than in immunocompetent C57BL/6 mice, consistent with more rapid tumor progression in severely immunodeficient hosts (48). Representative imaging on Day 1 showed no obvious difference in bioluminescence signal between the two groups, suggesting comparable baseline tumor burden after inoculation (**Figure 5C**). By Day 4, bioluminescence imaging showed more prominent tumor signal in the CD73-overexpressing group in the syngeneic setting (**Figure 5D**). All mice developed carcinomatosis by day 15, and tumors were harvested from all animals for immune analysis at that time point. Tumor-infiltrating immune cells analysis revealed a significant reduction in intratumoral CD8^+^ T cells in CD73 overexpressing tumor, whereas CD4^+^ T cells and CD11b^+^Gr-1^+^ myeloid cells were not significantly different between the groups (**Figure 5E**). These findings are consistent with our observations in human PDAC cohorts, in which CD73-high tumors showed reduced CD8^+^ T-cell infiltration, but not other immune components. In addition, CD8^+^ T cells showed significantly reduced the proliferation index co-cultured with CD73 overexpressing cells (**Figure 5F**). Collectively, these results suggest that CD73 associated tumor progression *in vivo* is strongly influenced by host immune context and is accompanied by reduced infiltrated CD8^+^ T cell infiltration and proliferative capacity.

### 3.7 PDAC patients with CD73 high expression tumor showed worse survival

**Figure 6:**
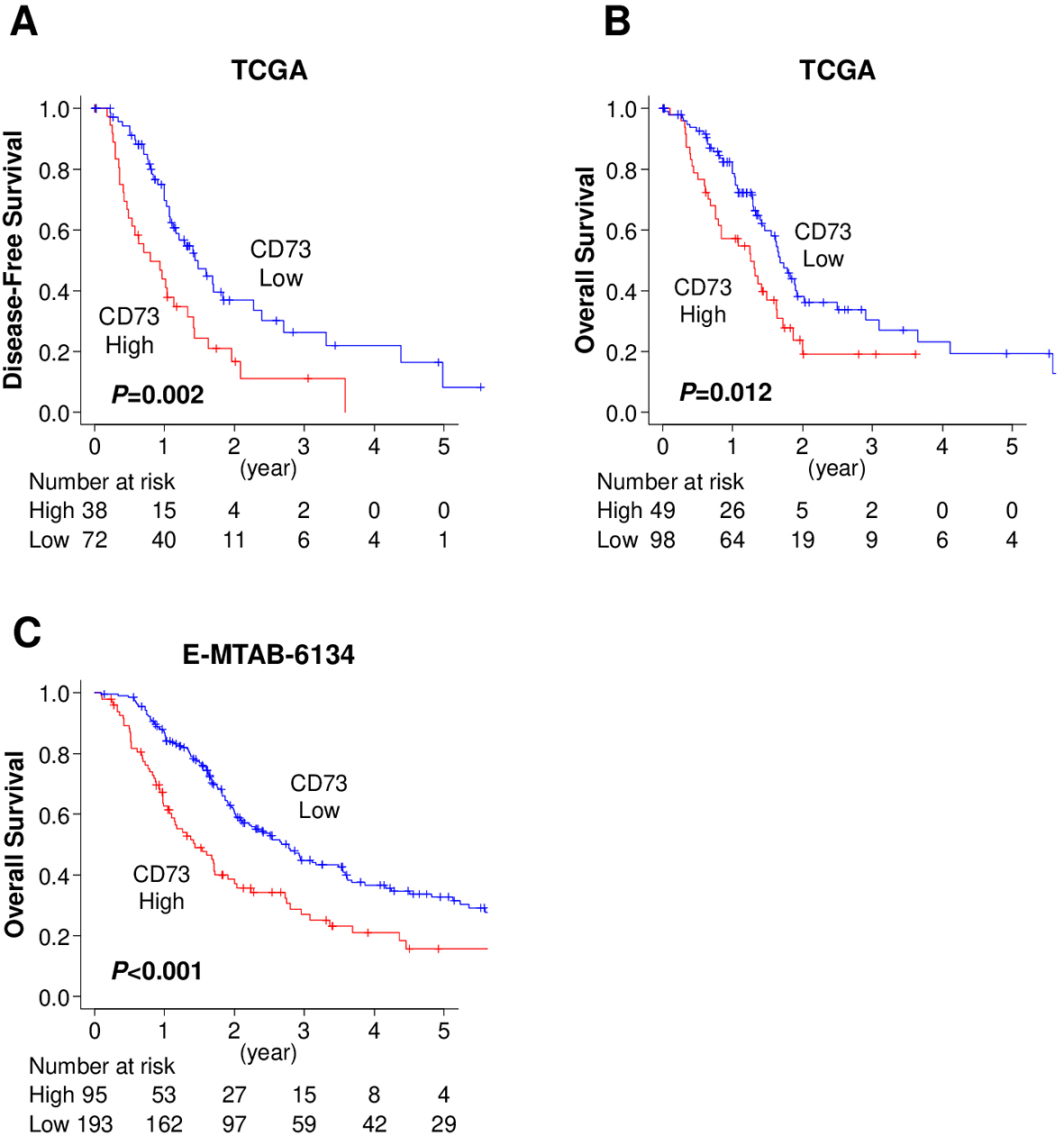
Pancreatic ductal adenocarcinoma (PDAC) patients with CD73 high expression tumor showed worse survival. Disease-Free **(A)** and Overall survival **(B)** by CD73 expression in TCGA PDAC cohort. **(C)** Overall survival by CD73 expression in E-MTAB-6134 cohort.

Lastly, we evaluated the prognostic significance of *CD73* gene expression in PDAC patient cohorts. In the TCGA dataset, patients with *CD73*-high PDAC exhibited significantly shorter disease-free survival and overall survival compared to *CD73*-low tumor (**Figures 6A, B**). This prognostic association was consistently reproduced in E-MTAB-6134 (**Figure 6C**). To further validate the robustness of this finding, we additionally analyzed the ICGC-CA cohort and confirmed that patients with *CD73*-high tumors also showed significantly worse survival in this independent cohort (**Supplementary Figure 5**). Across the examined datasets, elevated CD73 expression was consistently associated with unfavorable clinical outcomes, highlighting CD73 as a reproducible biomarker of aggressive tumor behavior in PDAC.

## 4 Discussion

**Figure 7:**
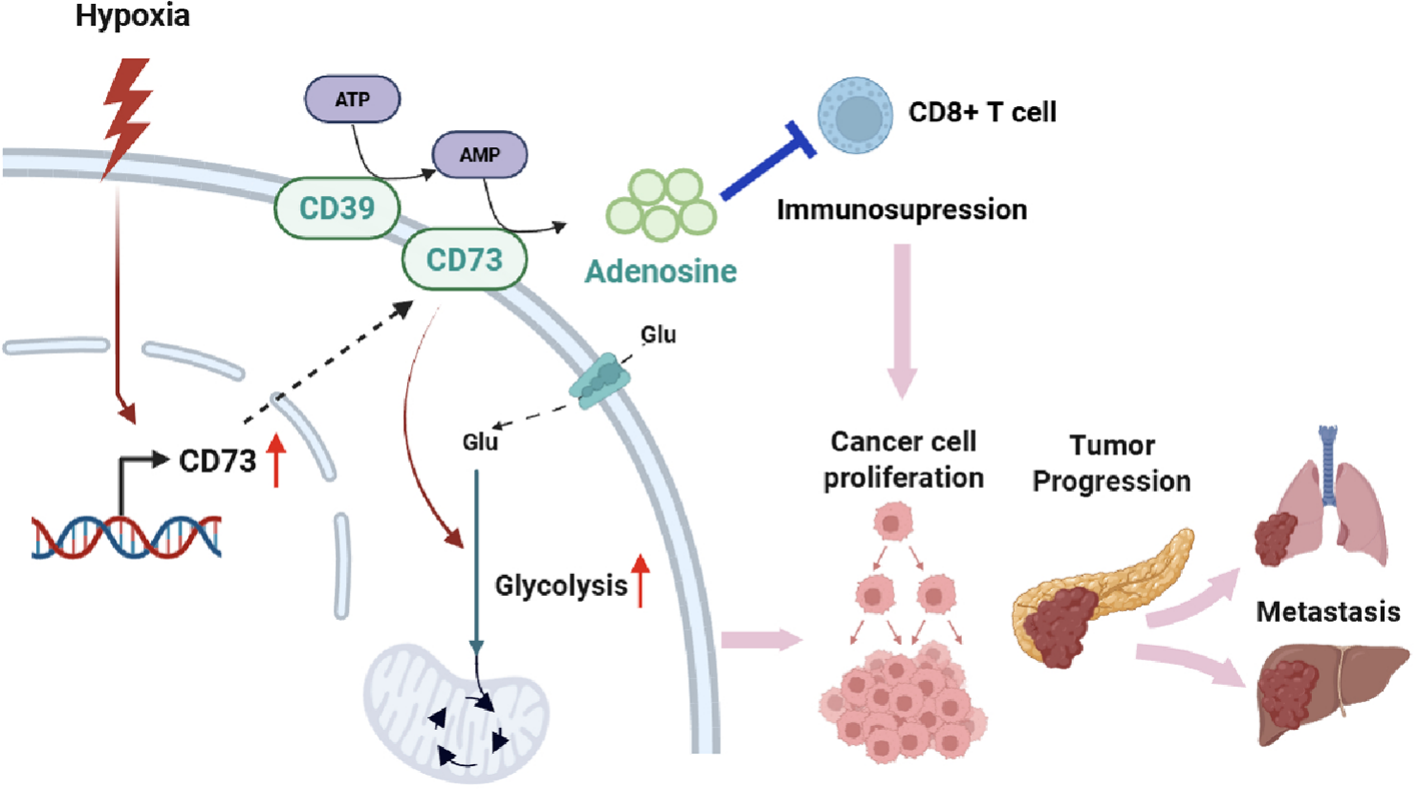
Proposed model for CD73 and its role in PDAC.

In this study, we found that CD73 was associated with hypoxia signaling, glycolysis-related metabolic phenotypes, cancer cell growth, and immune evasion in PDAC. Using transcriptomic datasets, *in vitro* functional assays, and *in vivo* syngeneic models, we observed CD73 expressions correlated with hypoxia, glycolysis related, and cell cycle gene programs. Hypoxic exposure increased CD73 mRNA expression in a subset of PDAC cell lines, and CD73 modulation affected glycolysis-related ECAR and cancer cell growth in a context-dependent manner. In human PDAC cohorts and syngeneic mouse models, high CD73 expression was associated with reduced CD8^+^ T cell infiltration and cytolytic activity. These findings support a model in which CD73 is linked to both metabolic and immunologic features of PDAC progression (**Figure 7**), while further mechanistic studies will be required to define direct causal relationships among these processes.

CD73 has previously been described as a hypoxia-responsive molecule, transcriptionally regulated by HIF-1α and HIF-2α through canonical hypoxia-responsive elements in the *CD73* promoter (4, 49). Consistent with these reports, we observed correlation of *CD73* and hypoxia gene signatures in PDAC across TCGA and E-MTAB-6134 datasets. Furthermore, 2 out of 3 examined PDAC cell lines cultured under hypoxic conditions exhibited induction of *CD73*. On the other hand, *CD73* mRNA expression was not significantly increased in AsPC-1 cells under hypoxic conditions. These findings suggest that hypoxia-mediated regulation of CD73 may occur in a context-dependent manner among PDAC cell lines and may be influenced by cell-intrinsic regulatory mechanisms. These results highlight the linkage between hypoxia and CD73 in PDAC and underscore its pathophysiologic relevance given the profoundly hypoxic nature of the PDAC tumor microenvironment.

Our integrative analysis also revealed an association between CD73 and glycolytic-related metabolic programs in PDAC. Enhanced glycolysis is one of the most well-established metabolic adaptations in PDAC, providing ATP, biosynthetic intermediates, and nucleotide precursors necessary for rapid cell proliferation (45, 50). GSEA from two independent patient cohorts consistently demonstrated correlation of glycolysis signatures and CD73 expression, suggesting that CD73-high tumors are transcriptionally associated with glycolysis-related programs. Functionally, Seahorse extracellular flux analysis showed that CD73 knockdown modestly but significantly reduced ECAR in PANC-1 cells. However, this effect was not clearly reproduced in AsPC-1 cells, suggesting that the metabolic impact of CD73 may be cell line dependent rather than universal across PDAC cells. Moreover, ECAR provides an indirect readout of extracellular acidification and glycolysis-related metabolic activity, but does not directly quantify glycolytic flux or glycolysis-related metabolites such as lactate, glucose-6-phosphate, or pyruvate. Therefore, our findings should be interpreted as suggestive evidence that CD73 may contribute to glycolysis-related metabolic regulation in a context-dependent manner. While CD73 has classically been studied as an ectoenzyme that generates extracellular adenosine, emerging evidence suggests that the CD73-adenosine axis can also support glycolytic reprogramming in cancer cells (31, 42). In gastric cancer, CD73 was shown to promote the Warburg effect in an enzyme activity-dependent manner, as pharmacologic inhibition of CD73 suppressed glycolysis and this effect was largely rescued by exogenous adenosine (42). Consistent with this concept, our findings suggest that CD73 may be linked to metabolic wiring in a subset of PDAC cells, although additional studies using metabolic profiling, lactate measurements, and recue or inhibition experiments will be required to define the precise conditions under which CD73 modulates glycolytic flux.

A key insight from our study is the observation that CD73-mediated glycolytic activation translates into accelerated cell-cycle progression and enhanced tumor proliferation. Glycolysis fuels nucleotide biosynthesis through the pentose phosphate pathway and supplies intermediates needed for lipid and amino-acid synthesis, thereby coupling metabolic reprogramming directly to S-phase entry (51). Across both PDAC cohorts analyzed, cell-cycle and G2/M checkpoint gene signatures were strongly correlated with CD73 expression. Functionally, ectopically expressed CD73 accelerated PDAC cell growth, while stable CD73 knockdown impaired proliferation. However, ectopically expressed CD73 did not enhance tumor growth in NSG mouse model. The discrepancy between the *in vitro* proliferation assay and the NSG survival experiment suggests that CD73-associated tumor progression *in vivo* is not solely determined by tumor cell-intrinsic proliferative activity. Although CD73 expression was associated with enhanced cell-cycle signatures and increased tumor cell growth *in vitro*, these effects were not accompanied by a significant survival difference in immunodeficient NSG mice. Therefore, the contribution of CD73-mediated proliferative programs to *in vivo* tumor progression may be limited, and our findings do not support a strong conclusion that enhanced proliferative activity alone explains the observed survival phenotype.

Beyond metabolic functions, our results emphasize the critical role of CD73 in shaping the immune microenvironment in PDAC, consistent with prior work demonstrating that CD73-generated adenosine suppresses T cell activity (3, 12). In human PDAC cohorts, CD73-high tumors exhibited significantly reduced CD8^+^ T cell infiltration. Additional transcriptomic analyses further demonstrated significantly lower CD8 T cell and cytolytic activity scores in CD73-high tumors, whereas exhaustion-related signatures were not significantly increased. These findings suggest that CD73-high PDAC is characterized primarily by reduced cytotoxic T cell infiltration and activity rather than a clearly exhaustion-dominant immune phenotype. This interpretation is consistent with the emerging concept that the balance between effector T cells (Teff) and regulatory T cells (Treg), often represented by the Teff/Treg ratio, is a critical determinant of anti-tumor immune competence and therapeutic responsiveness in PDAC (52). A reduced cytotoxic T cell component in CD73-high tumors may therefore reflect a shift toward an immunologically unfavorable T cell balance, although our bulk RNA-seq and limited flow cytometry analyses were not sufficient to directly quantify the Teff/Treg ratio. However, because exhaustion-related signatures were inferred from bulk RNA-seq data, they are influenced by T cell abundance and cannot determine the functional state of residual CD8^+^ T cells at the single-cell level. In syngeneic mouse models, CD73 overexpression accelerated tumor progression and decreased intratumoral CD8^+^ T cells, whereas these effects were absent in immunodeficient NSG mice. Our findings suggest that host immune factors may play an important role in mediating the tumor-promoting effects of CD73.

Therapeutically, these findings support the relevance of the CD73–adenosine axis as a potential target in PDAC. CD73-targeting strategies, including anti-CD73 antibodies and small-molecule inhibitors, are currently being evaluated in PDAC and other solid tumors (53–55). However, the clinical efficacy of CD73 blockade is likely to depend on biomarker-driven patient selection and rational combination strategies. CD73 expression can arise from tumor cells, stromal cells, and immune cells, and the biological consequences of CD73 may vary according to tumor hypoxia, myeloid inflammation, cytotoxic T cell infiltration, and broader adenosine pathway activity. Therefore, CD73 expression alone may not be sufficient as a predictive biomarker. Integrated biomarkers incorporating CD73 expression, CD39/CD73 pathway activity, hypoxia-related programs, and cytotoxic immune infiltration may be required to identify patients most likely to benefit from CD73-targeted therapy. Given the highly immunosuppressive and heterogeneous nature of PDAC, CD73 blockade combined with chemotherapy, immune checkpoint blockade, myeloid-targeting strategies, or metabolic interventions may be options to achieve clinically meaningful anti-tumor effects.

Several limitations should be acknowledged in this study. First, immune cell infiltration was inferred computationally from bulk RNA-seq data using the CIBERSORT algorithm. These estimations do not represent direct measurements of immune cell populations and may be influenced by tumor purity and transcriptomic heterogeneity. Therefore, validation using orthogonal approaches such as flow cytometry, immunohistochemistry, spatial transcriptomics, or single-cell RNA sequencing will be required in future studies. Second, although hypoxia-induced CD73 mRNA upregulation was observed in two of three PDAC cell lines, CD73 protein expression under hypoxic conditions was not directly assessed. While the observed transcriptional changes are consistent with previously reported HIF-1α-dependent regulation of CD73 (4), future studies will be required to determine whether these changes are accompanied by corresponding alterations in CD73 protein abundance. Moreover, our qPCR analyses used *ACTB* as a single reference gene. Although this approach is supported by prior hypoxia-related pancreatic cancer studies, multi-gene normalization strategies may provide greater robustness under hypoxic stress conditions. Third, although increased cell numbers were observed in association with CD73 expression, the present study did not distinguish between enhanced proliferation and reduced apoptosis. Future studies employing proliferation- and apoptosis-specific assays will be required to clarify the underlying mechanisms. In addition, adenosine generated by CD73 may promote tumor growth through A2A receptor-dependent signaling pathways, including cAMP/PKA signaling, which warrants further investigation. Furthermore, experiments using glycolytic inhibitors such as 2-deoxyglucose may help determine whether enhanced glycolytic activity is mechanistically required for the growth-promoting effects of CD73. Fourth, while our results link CD73 to both glycolytic activity and reduced CD8^+^ T cell infiltration, the causal mechanisms connecting these processes were not fully dissected. Additional rescue and pathway-blocking experiments will be required to determine whether CD73 directly and causally links hypoxia, glycolytic regulation, cell cycle progression, and immune suppression in PDAC. In addition, our flow cytometric analysis used a limited myeloid panel, and the CD11b^+^Gr-1^+^ population could not be further subdivided into monocytic MDSCs, granulocytic MDSCs, neutrophils, or macrophage subsets. More detailed immune profiling will be required in future studies. Finally, our *in vivo* findings were primarily obtained in a Panc02 syngeneic model, and validation in additional PDAC models will be necessary. Accordingly, the translational significance of CD73 as a therapeutic target in PDAC warrants further investigation.

In summary, our study expands the current understanding of CD73 in PDAC by showing that, in addition to its established immunosuppressive role, CD73 is linked to hypoxia-associated metabolic rewiring and proliferative programs. These findings support the relevance of CD73 as a potential biomarker and therapeutic target in PDAC and provide a rationale for further mechanistic studies of the CD73-adenosine axis.

## Data Availability

The link for TCGA data is as follows; https://www.cbioportal.org/. The link for E-MTAB-6134 is as follows; https://www.ebi.ac.uk/biostudies/arrayexpress/studies/E-MTAB-6134.
