# Supplementary material for: CD73 is associated with glycolysis related metabolic programs and CD8+ T cell suppression in pancreatic ductal adenocarcinoma
Source: Front Immunol. 2026 Jul 27;17:1873813. doi: 10.3389/fimmu.2026.1873813 (PMC13454112; doi:10.3389/fimmu.2026.1873813)
Supplement: Supplementary file 1 [file Image1.pdf]

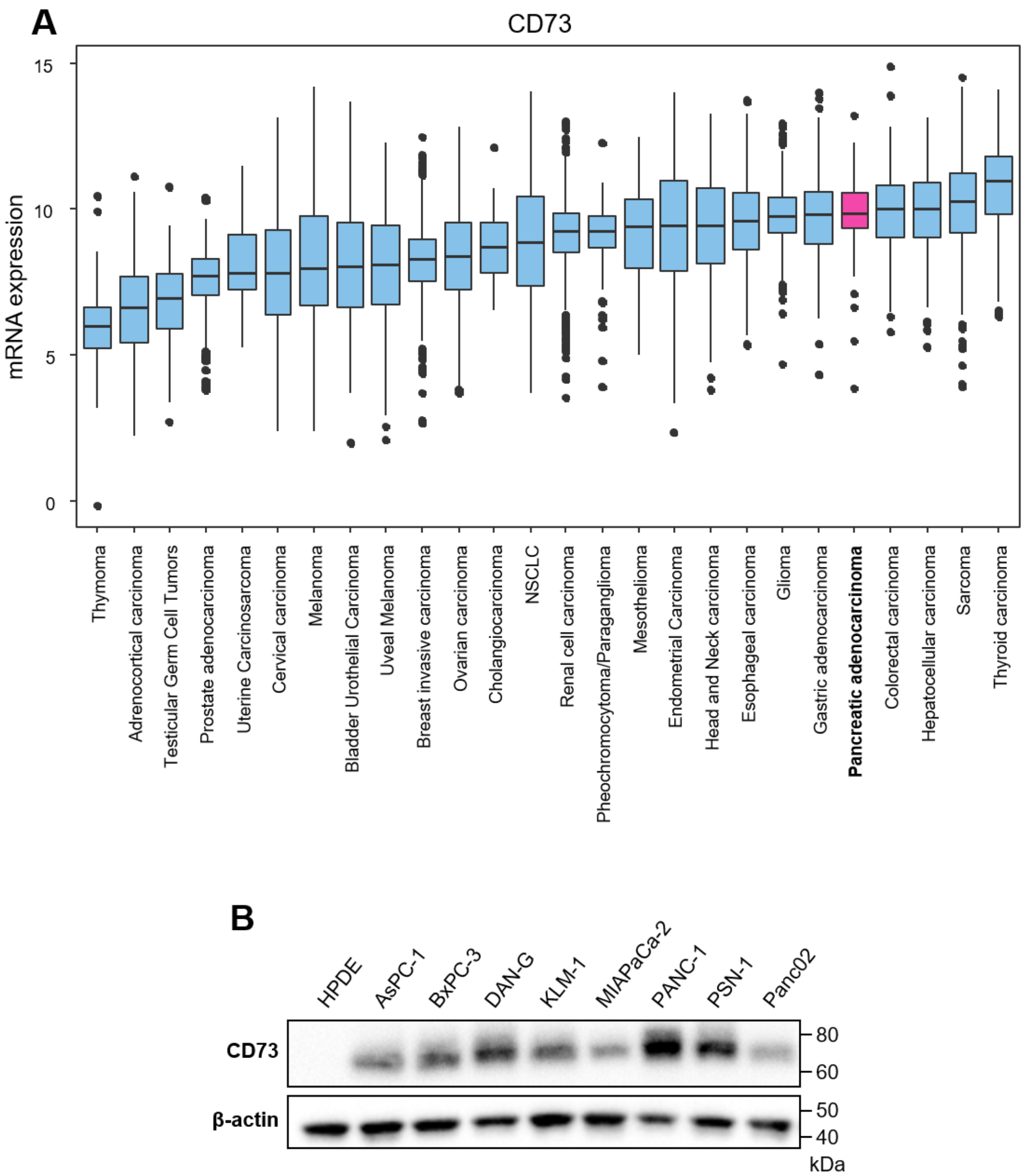

Figure S1: CD73 is highly expressed in pancreatic ductal adenocarcinoma (PDAC). (A) Pan-cancer transcriptomic profiles obtained from the TCGA database. (B) Protein expression of CD73 in normal pancreatic duct epithelial cell line (HPDE) and PDAC cell lines (AsPC-1, BxPC-3, DAN-G, KLM-1, MIAPaCa-2, PANC-1, PSN-1, and Panc02).

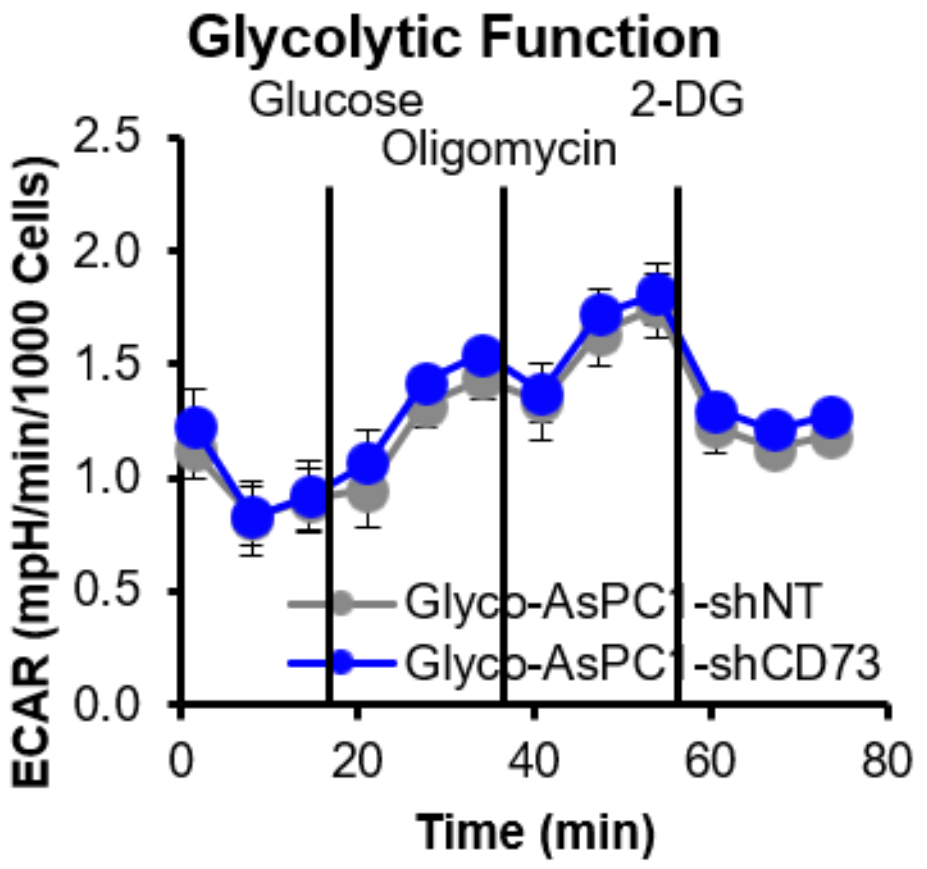

Figure S2: Glycolysis function change in CD73 knockdown AsPC-1 cells by Seahorse assay (n=6).

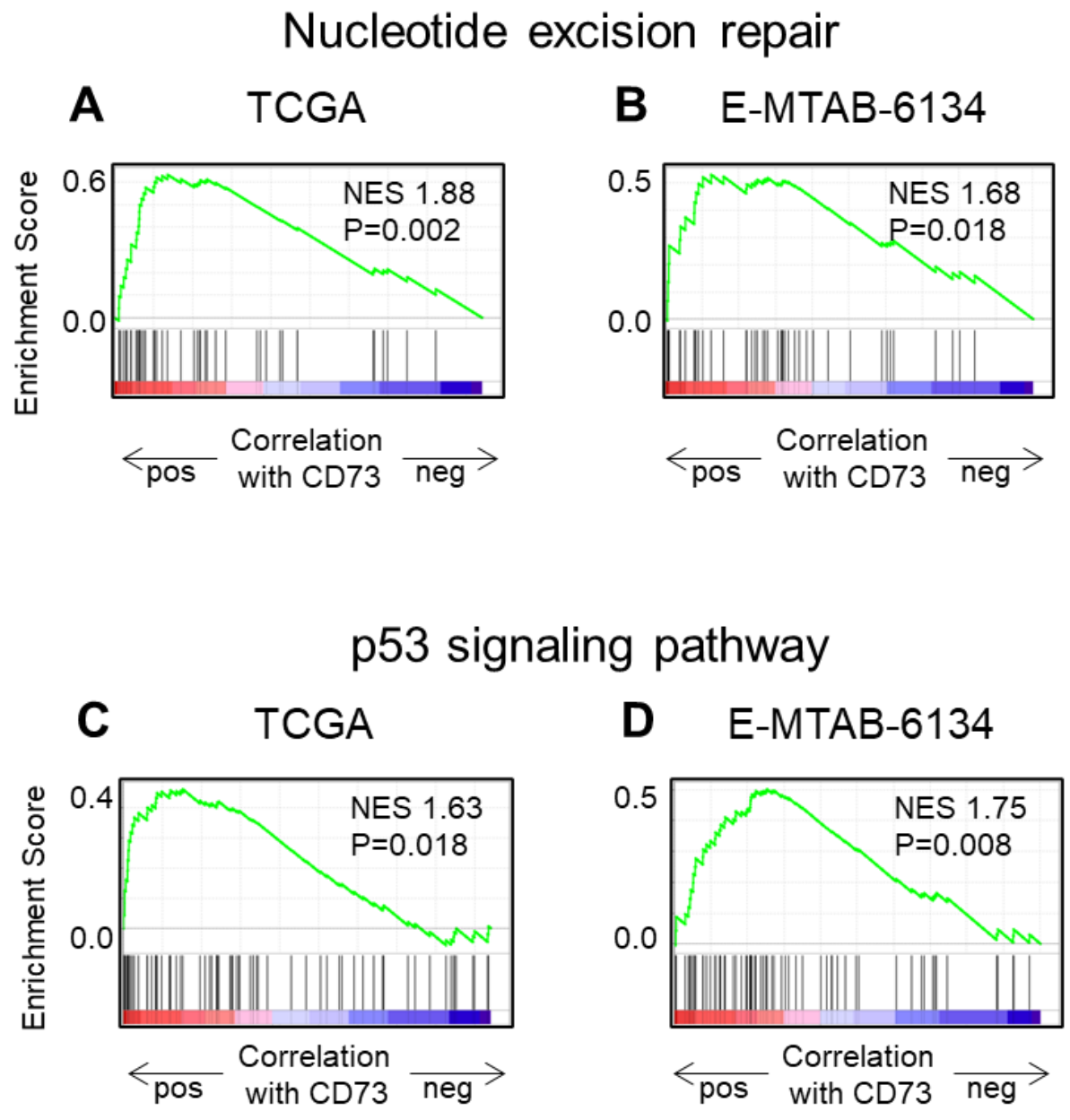

Figure S3: CD73 expression was correlated with DNA repair genes in PDAC. GSEA of nucleotide excision repair in (A) TCGA and (B) E-MTAB-6134 and p53 signaling pathway in (C) TCGA and (D) E-MTAB-6134.

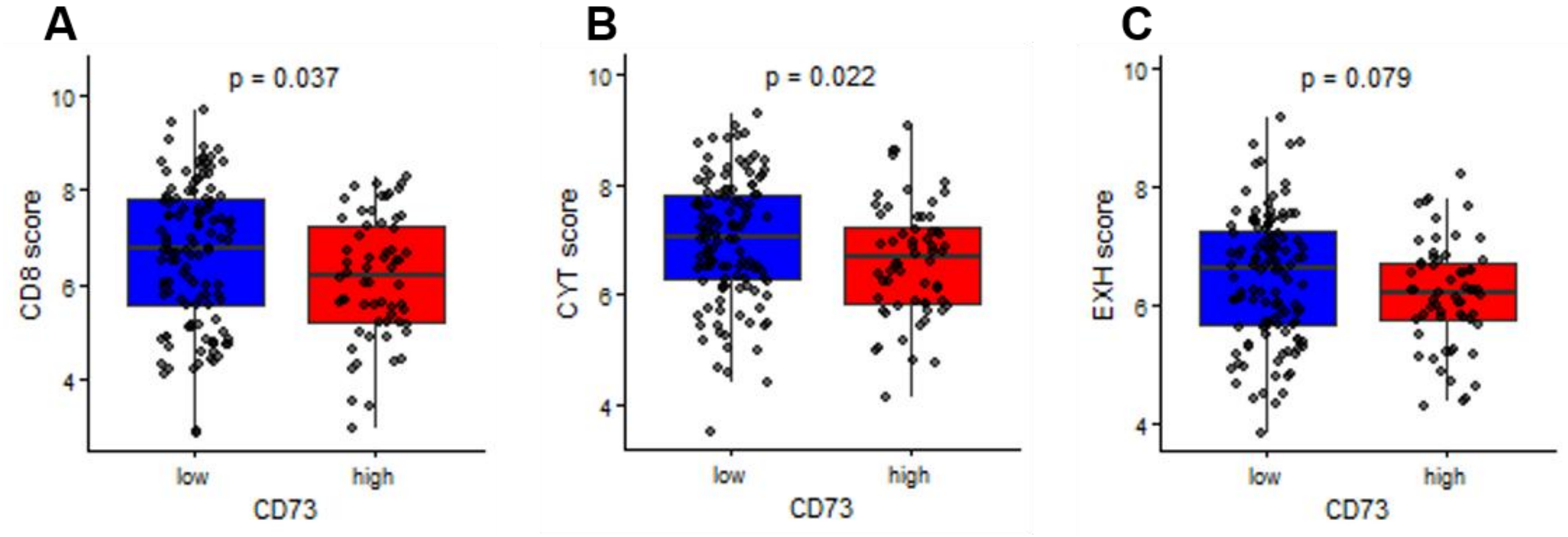

Figure S4. Comparison of immune-related signature scores between CD73-high and CD73-low tumors.

(A) CD8 T cell signature score, (B) Cytolytic activity (CYT) score, and (C) Exhaustion (EXH) score.

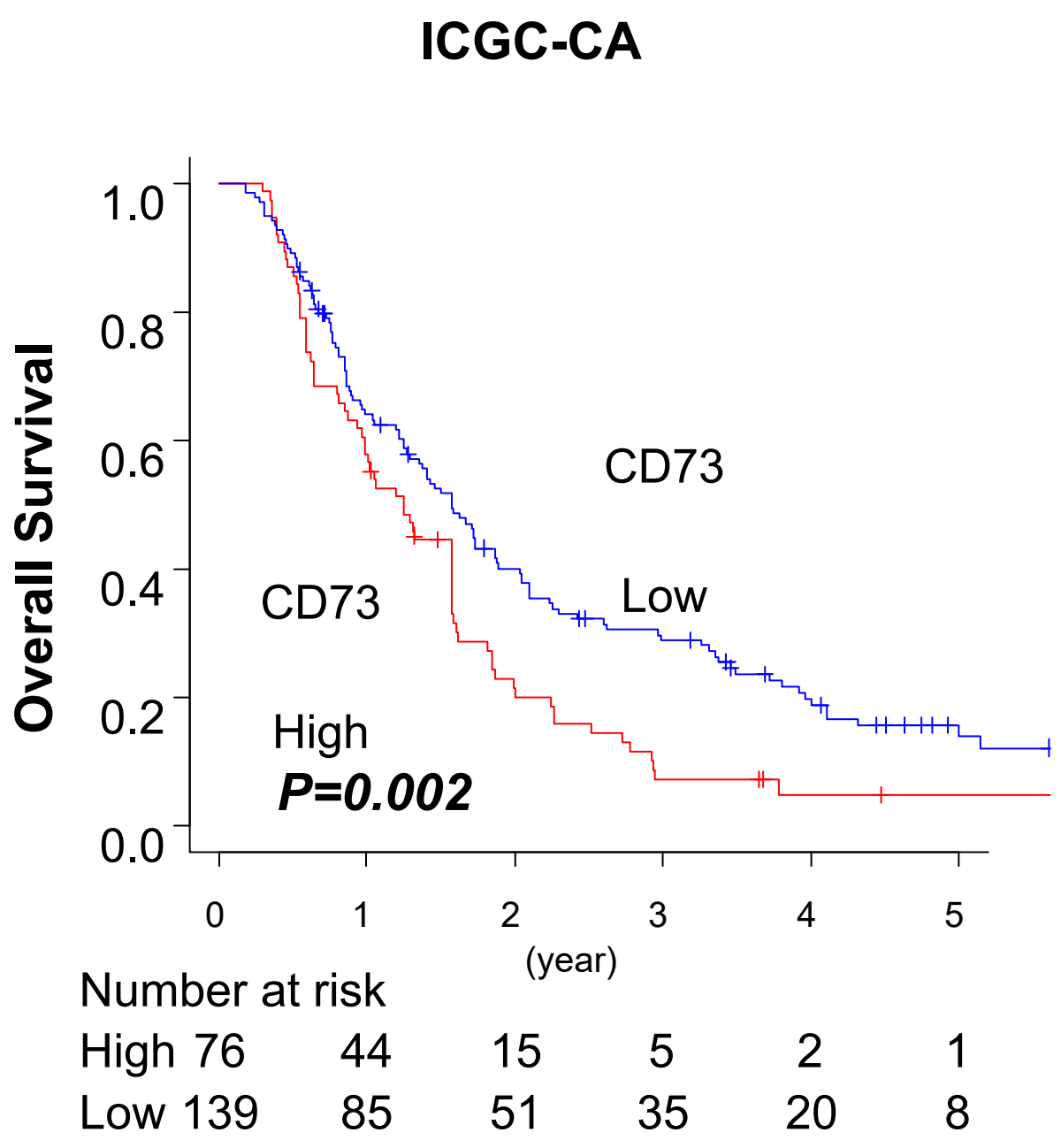

Figure S5: Overall survival by CD73 expression in ICGC-CA PDAC cohort.
