# Supplementary material for: CD73 is associated with glycolysis related metabolic programs and CD8+ T cell suppression in pancreatic ductal adenocarcinoma
Source: Front Immunol. 2026 Jul 27;17:1873813. doi: 10.3389/fimmu.2026.1873813 (PMC13454112; doi:10.3389/fimmu.2026.1873813)

Fig. 1C

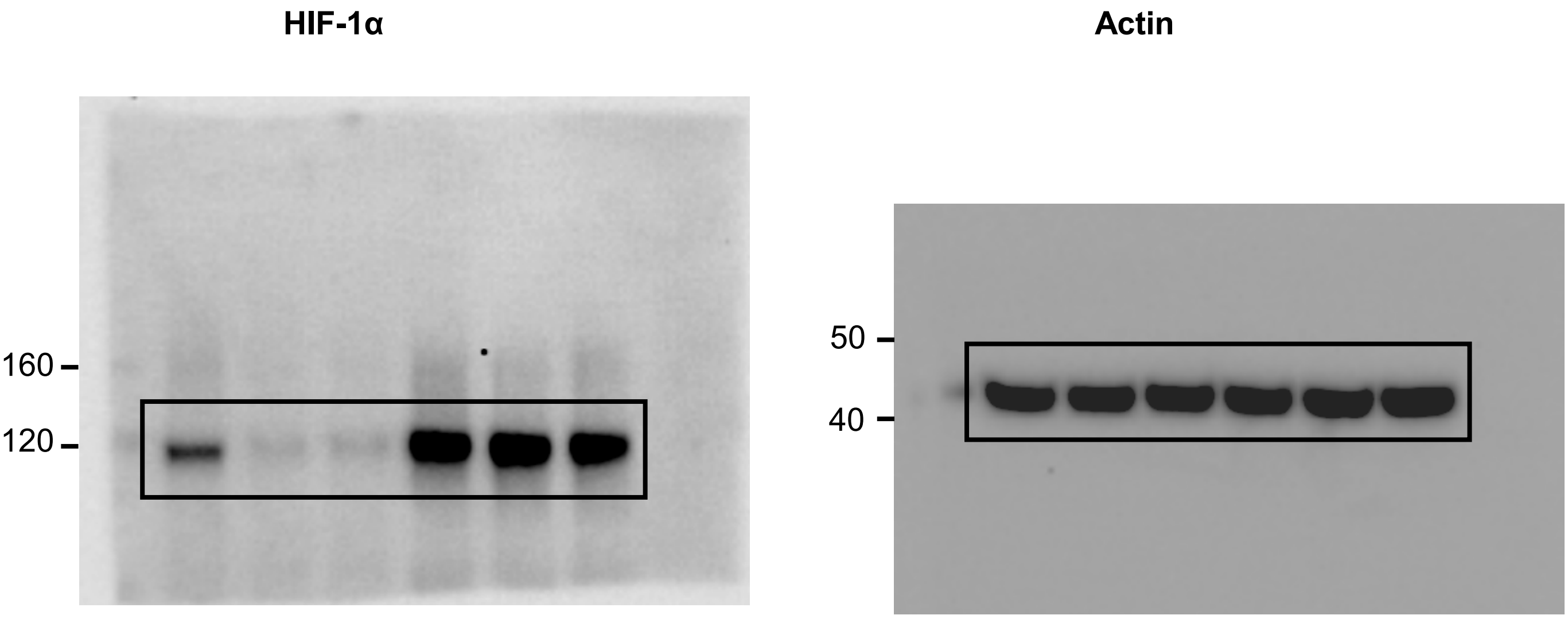

Fig. 2C

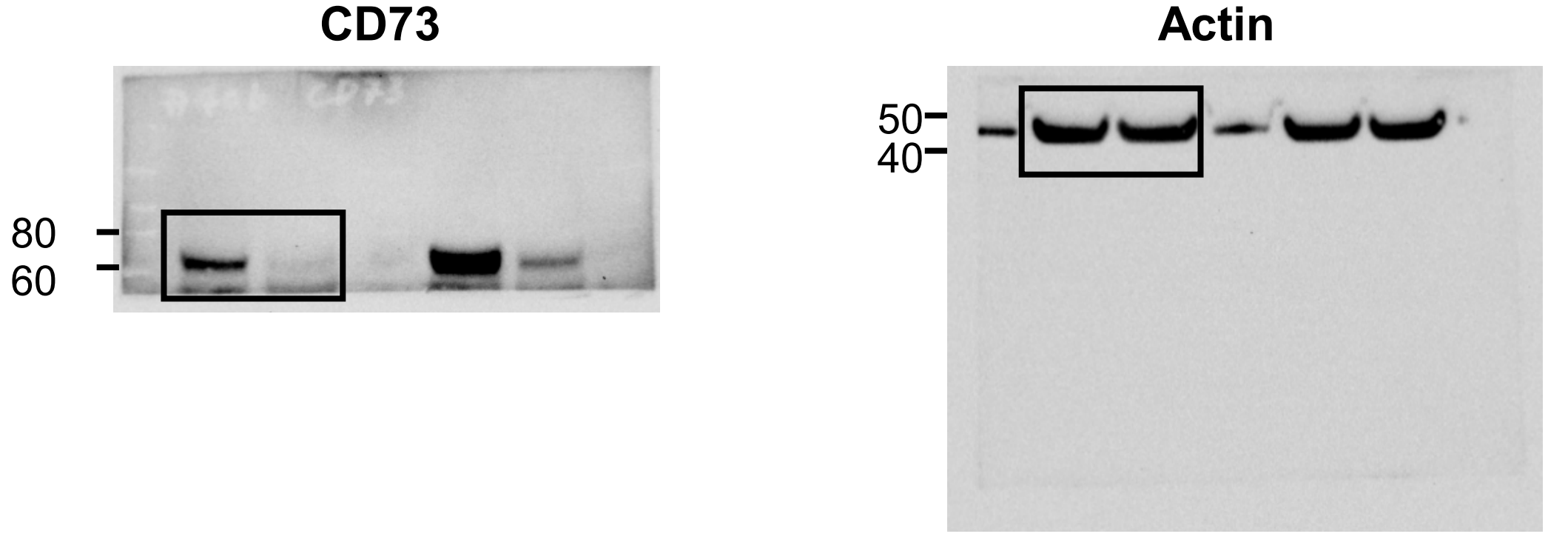

Fig. 3E

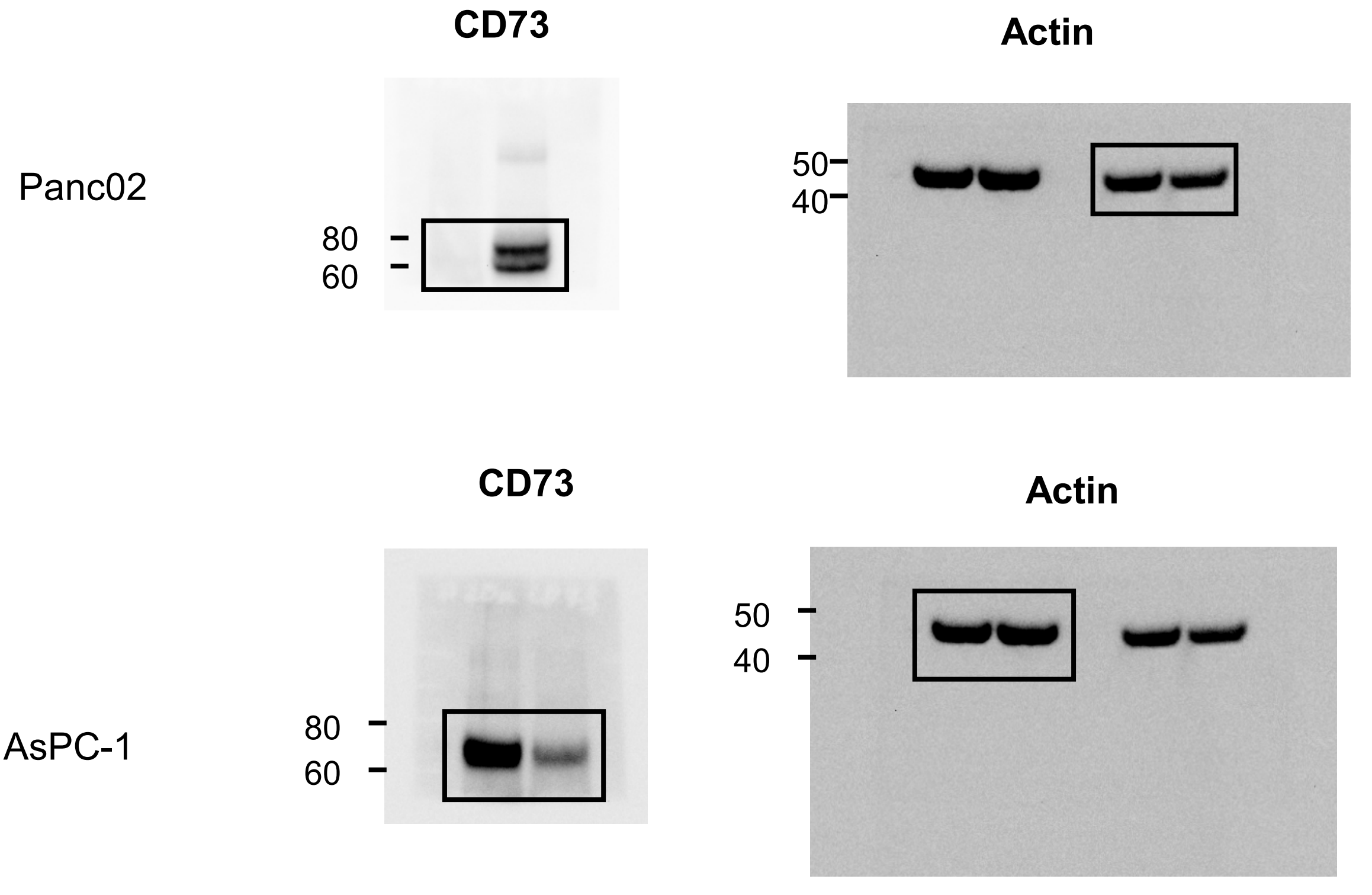

Fig. S1B

CD73

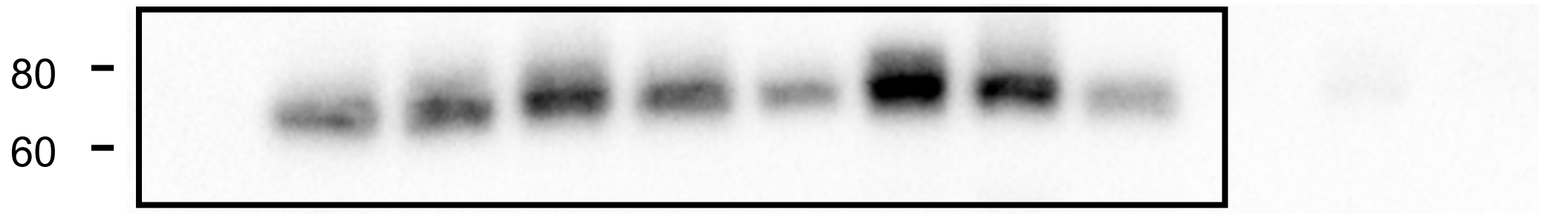

β-actin

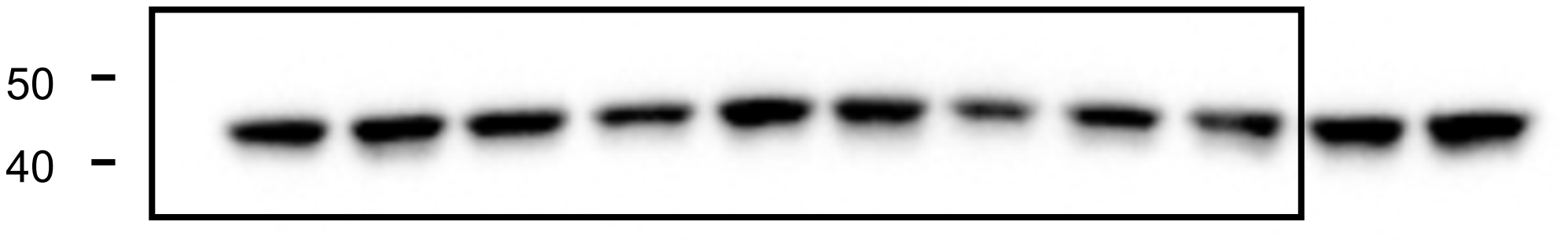

Supplement: Supplementary file 2 [file Image2.pdf]
