# Supplementary material for: CD73 is associated with glycolysis related metabolic programs and CD8+ T cell suppression in pancreatic ductal adenocarcinoma
Source: Front Immunol. 2026 Jul 27;17:1873813. doi: 10.3389/fimmu.2026.1873813 (PMC13454112; doi:10.3389/fimmu.2026.1873813)
Supplement: Supplementary file 3 [file Table1.pdf]

Table S1 qPCR Primer sequence

Gene	F	R
CD73	GGTCAAATGTGCCTCCAAAG	ATTGATGAACGCAACAATGG
GLUT1	CAGATAGGACATCCAGGGTAGC	GGTTGTGCCATACTCATGACC
PDK1	CACCAAGACCTCGTGTTGAG	AGACGTGATATGGGCAATCC
ACTB	GTGGCCGAGGACTTTGATTG	CCTGTAACAACGCATCTCATATT
